# DNA methylation of CpG sites in the chicken *KLF7* promoter and Exon 2 in association with mRNA expression in abdominal adipose tissue and blood metabolic indicators

**DOI:** 10.1186/s12863-020-00923-6

**Published:** 2020-10-14

**Authors:** Zhiwei Zhang, Cunxi Nie, Yuechan Chen, Yanzhe Dong, Tao Lin

**Affiliations:** 1 School of Medicine, Shihezi University, No. 59 Beier Road, Shihezi, Xinjiang, 832000 P. R. China; 2 College of Animal Science and Technology, Shihezi university, Shihezi, 832000 China; 3 First Affiliated Hospital of School of Medicine, Shihezi University, Shihezi, 832000 China

**Keywords:** Chicken, Adipose tissue, DNA methylation, Blood metabolic indicators, Krüppel-like factor 7

## Abstract

**Background:**

Our previous study found that chicken KLF7 was an important regulator in formation of adipose tissue. In the present study, we analyzed the association for DNA methylation in chicken *KLF7* with its transcripts of abdominal adipose tissue and blood metabolic indicators.

**Results:**

The *KLF7* transcripts of the adipose tissue of Chinese yellow broilers were associated with age (F = 6.67, *P* = 0.0035). In addition, the *KLF7* transcripts were negatively correlated with blood glucose levels (r = − 0.61841, *P* = 0.0140). The DNA methylation levels of 26 CpG loci in the chicken *KLF7* promoter and Exon 2 were studied by Sequenom MassArray. A total of 22 valid datasets were obtained. None of them was significantly different in relation to age (*P* > 0.05). However, the DNA methylation levels in the promoter were lower than those in Exon 2 (T = 40.74, *P* < 0.01). Correlation analysis showed that the DNA methylation levels of PCpG6 and E2CpG9 were significantly correlated with *KLF7* transcripts and blood high-density lipoprotein levels, respectively, and many CpG loci were correlated with each other (*P* < 0.05). The methylation data were subjected to principal component analysis and factor analysis. The six principal components (z1–z6) were extracted and named Factors 1–6, respectively. Factor analysis showed that Factor 1 had a higher load on the loci in the promoter, and Factors 2–6 loaded highly on quite different loci in Exon 2. Correlation analysis showed that only z1 was significantly correlated to *KLF7* transcripts (*P* < 0.05). In addition, an established regression equation between z1 and *KLF7* transcripts was built, and the contribution of z1 to the variation on *KLF7* transcripts was 34.29%.

**Conclusions:**

In conclusion, the *KLF7* transcripts of chicken abdominal adipose tissue might be inhibited by DNA methylation in the promoter, and it might be related to the DNA methylation level of PCpG6.

## Background

Krüppel-like factor 7 (KLF7), also known as ubiquitous KLF (UKLF), is a member of the KLFs, which are characterized by three Cys_2_-His_2_ zinc fingers at the C-terminus [1]. The *KLF7* gene is highly conserved among animals, especially mammalian species and birds.

Reports on mammalian specie showed that KLF7 played an important role in the differentiation of neuroectodermal and mesodermal cell lineages in vitro [2]. Gene targeting studies in mice implicated that *KLF7* was involved in nervous system development [3, 4], and mice without KLF7 activity displayed hypoplastic olfactory bulbs which lack peripheral innervation [5].

KLF7 had been reported as a key regulator of human obesity [6], diabetes mellitus type 2 (T2DM) [7, 8], and blood disease [9]. In addition, *KLF7* was an oncogene in several kinds of cancers, including gastric cancer [10], lung adenocarcinoma [11], glioma [12] and oral squamous cell carcinoma [13]. Additionally, Genetics analysis showed that *KLF7* was associated with self-rated health [14] and neurodevelopmental disorders [15] in human. Integrative genomic studies suggested that KLF7 might be one of the core factors that regulate cardiovascular diseases [16].

Our previous study in chickens showed that the number of *KLF7* transcripts of abdominal fat tissue was greater in lean broilers than in fat broilers, and KLF7 promotes the proliferation of chicken preadipocytes and inhibits their differentiation in vitro [17]. One SNP (c. A141G) in the *KLF7* coding sequence might be a molecular marker for the selection of blood very-low-density lipoprotein and abdominal fat content in broilers [18]. DNA methylation is a very well-studied epigenetic phenomenon that typically correlates with gene silencing [19]. The DNA methylation in *KLF7* is reported to be associated with the occurrence and development of gastric cancer in humans [20, 21]. However, the effect of *KLF7* DNA methylation in the adipose tissue of birds has not been reported.

Adipose tissue is an important site for lipid storage, energy homeostasis, and insulin sensitivity in human being and animals [22]. The previous study in vitro showed that overexpression of KLF7 might induce the development of T2DM via serval ways, including suppression of insulin secretion, insulin sensitivity and adipogenesis [7, 8]. However, whether the DNA methylation of *KLF7* in the adipose tissue had associations with its expression and the blood metabolic indicators, including the levels of glucose (Glu), phospholipids (PL), total cholesterol (TC), triglyceride (TG), low-density lipoprotein (LDL) and high-density lipoprotein (HDL), which were correlated with the T2DM and obesity, is still unclear. In the present study, the associations for DNA methylation in the chicken *KLF7* gene with its expression and fasting blood metabolic indicators were studied. The results might improve the understanding of *KLF7* expression and function in adipose tissue.

## Results

### *KLF7* mRNA expression of chicken adipose tissues

**Fig. 1:**
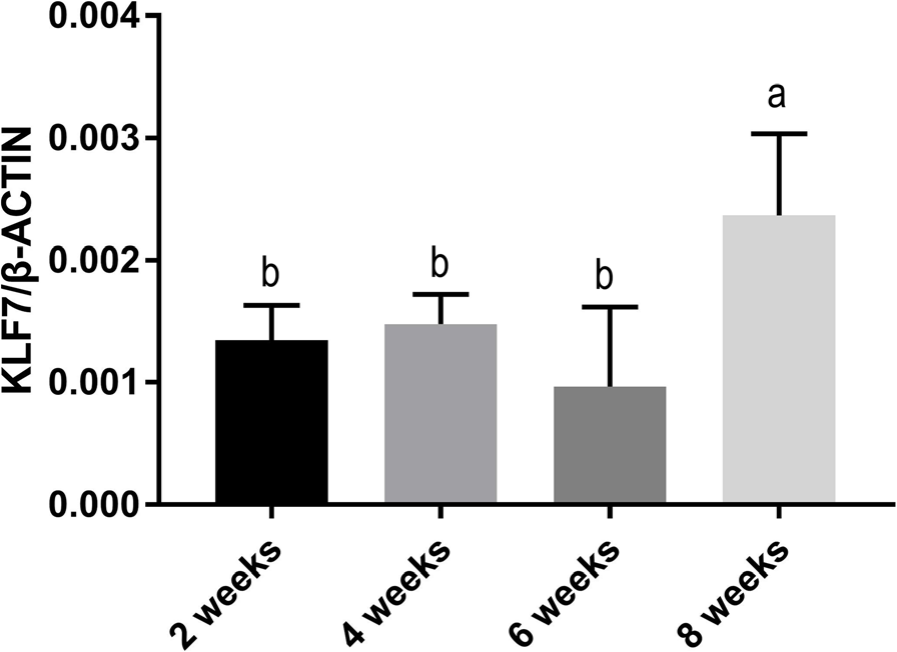
The mRNA expression pattern of *KLF7* in chicken abdominal fat tissue. *β-ACTIN* was used as an internal control. The diagram shows the relative quantification of *KLF7* expression. Error bars indicate the standard deviations from biological replicates (*n* ≥ 4). The different lowercase letters above columns indicate significant difference in various ages (Duncan’s multiple tests, *P* < 0.05)

The expression of *KLF7* mRNA in the abdominal adipose tissue of Chinese fast-growing yellow broilers at the age of 2, 4, 6, and 8 weeks was studied by real-time PCR. The results showed that *KLF7* mRNA was expressed in all the tissues studied. The *KLF7* transcripts data conformed to a normal distribution (Shapiro–Wilk test; W = 0.957301, *P* = 0.4635). ANOVA analysis showed that *KLF7* transcripts were significantly associated with age (F = 6.67, *P* = 0.0035), and at the age of 8 weeks, the level of *KLF7* transcripts was significantly greater than that at the age of 2, 4, and 6 weeks (*P* < 0.05, Fig. 1).

### The level of blood metabolic indicators during the development and growth of broilers

**Fig. 2:**
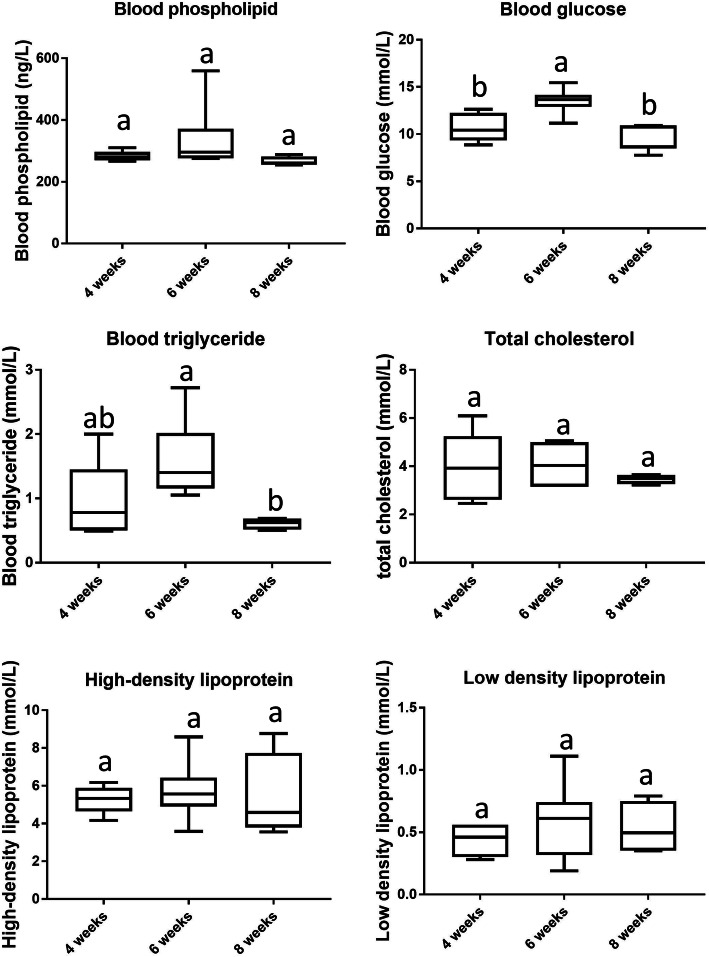
Levels of glucose (Glu), phospholipids (PL), total cholesterol (TC), triglyceride (TG), low-density lipoprotein (LDL), and high-density lipoprotein (HDL) in the blood of fasting Chinese fast-growing yellow broilers. The different lowercase letters above columns indicate significant difference in various ages (Duncan’s multiple tests, *P* < 0.05)

The levels of Glu, PL, TC, TG, LDL and HDL in the blood of fasting Chinese fast-growing yellow broilers at the age of 4, 6, and 8 weeks were studied by spectroscopy. ANOVA or Kruskal–Wallis analysis showed there was no significant difference in the levels of PL, TC, LDL, and HDL in chicken blood among the ages of 4, 6, and 8 weeks (*P* > 0.05, Table 1). However, blood glucose levels in 6-week-old broilers were significantly greater than those in 4- and 8-week-old broilers (*P* < 0.05, Fig. 2), and the blood TG contents of 8-week-old broilers were significantly lower than those of 6-week-old broilers (P < 0.05, Fig. 2). Spearman’s correlation analysis showed that *KLF7* transcripts were significantly negatively correlated with fasting blood glucose concentration (r = − 0.61841, *P* = 0.0140), and there was no significant association between *KLF7* transcripts and other blood parameters (*P* > 0.05).

**Table 1:** Datasets characteristics of chicken blood metabolic indicators

Dataset	N ^a^	Shapiro-Wilk test		Difference test among ages	
PL	15	W = 0.504656	*P* < 0.0001	H = 5.1574	*P* = 0.0759
GLU	15	W = 0.970623	*P* = 0.8672	F = 8.24	*P* = 0.0056
TG	15	W = 0.863964	*P* = 0.0275	H = 7.3490	*P* = 0.0254
TC	15	W = 0.940254	*P* = 0.3856	F = 0.43	*P* = 0.6586
HDL	15	W = 0.864245	*P* = 0.0278	H = 1.1558	*P* = 0.5611
LDL	15	W = 0.916808	*P* = 0.1722	F = 0.52	*P* = 0.6077

Note: ^a^ represents the number of samples analysed

### CpG density in the genomic region of chicken *KLF7*

**Fig. 3:**
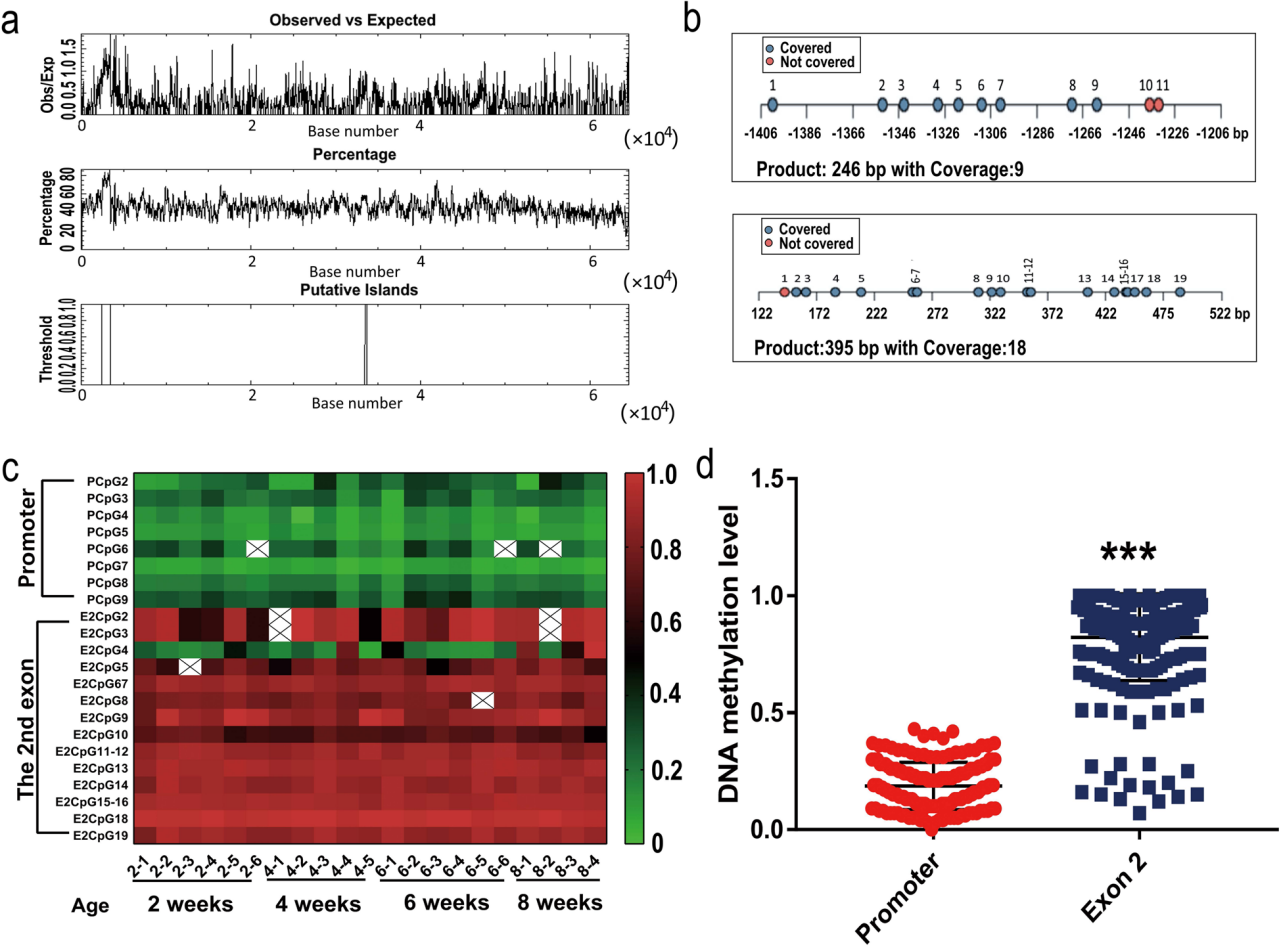
DNA methylation analysis of chicken *KLF7* in adipose tissues. **a**. The CpG density of the genomic region of chicken *KLF7* analyzed by CpGplot (Version 6.6.0). **b**. The CpG loci analyzed in the promoter and Exon 2. **c**. The levels of DNA methylation of CpG loci analyzed. **d**. The comparison of DNA methylation levels between the promoter and Exon 2. Asterisks indicate a significant difference between the promoter and Exon 2 (Student’s *t* test, ***P < 0.001)

The CpG density analysis showed that there were two CpG islands in the genomic region of chicken *KLF7*. One CpG island, with a sequence interval of − 1156 to − 164 bp upstream of the *KLF7* translation initiation site, was about 993 bp in length and located in the promoter and the other was located in Exon 2. The sequence interval between 254 bp to 523 bp after the first nucleotide of Exon 2, was about 269 bp in length (Fig. 3a).

### DNA methylation of chicken *KLF7* in adipose tissues

Sequenom MassArray was used to study DNA methylation in CpG-rich sequences of chicken *KLF7*. In the promoter region, the DNA methylation levels of nine CpG loci in the sequence interval from − 1452 bp to − 1206 bp upstream of the translation initiation site were studied (Fig. 3b), and eight valid datasets were obtained (Fig. 3c). The data were distributed normally (*P* > 0.05, Table 2). ANOVA analysis found no significant difference in the DNA methylation level of any of these eight loci among the ages of 2, 4, 6, and 8 weeks (*P* > 0.05).

**Table 2:** Datasets characteristics of DNA methylation of chicken *KLF7* in abdominal adipose tissues

Dataset	N ^a^	Shapiro-Wilk test		Difference test among ages	
**PCpG2**	21	W = 0.963142	*P* = 0.5815	F = 0.74	*P* = 0.5405
**PCpG3**	21	W = 0.940649	*P* = 0.2244	F = 0.10	*P* = 0.9566
**PCpG4**	21	W = 0.935328	*P* = 0.1760	F = 0.42	*P* = 0.7418
**PCpG5**	21	W = 0.95307	*P* = 0.3888	F = 0.35	*P* = 0.7874
**PCpG6**	18	W = 0.944124	*P* = 0.3404	F = 0.22	*P* = 0.8776
**PCpG7**	21	W = 0.96436	*P* = 0.6079	F = 0.49	*P* = 0.6906
**PCpG8**	21	W = 0.964176	*P* = 0.6039	F = 0.34	*P* = 0.7965
**PCpG9**	21	W = 0.977153	*P* = 0.8795	F = 0.07	*P* = 0.9753
**E2CpG2**	19	W = 0.792999	*P* = 0.0009	H = 3.4737	*P* = 0.3242
**E2CpG3**	19	W = 0.792999	P = 0.0009	H = 3.4737	P = 0.3242
**E2CpG4**	21	W = 0.803609	*P* = 0.0007	H = 5.5746	*P* = 0.1342
**E2CpG5**	20	W = 0.937047	*P* = 0.2107	F = 0.19	*P* = 0.9050
**E2CpG6–7**	21	W = 0.947677	*P* = 0.3077	F = 0.17	*P* = 0.9121
**E2CpG8**	20	W = 0.943714	*P* = 0.2815	F = 0.31	*P* = 0.8190
**E2CpG9**	21	W = 0.948257	*P* = 0.3156	F = 0.61	*P* = 0.6192
**E2CpG10**	21	W = 0.93046	*P* = 0.1406	F = 1.38	*P* = 0.2836
**E2CpG11–12**	21	W = 0.921137	*P* = 0.0914	F = 2.82	*P* = 0.0703
**E2CpG13**	21	W = 0.921203	*P* = 0.0917	F = 0.42	*P* = 0.7419
**E2CpG14**	21	W = 0.969337	*P* = 0.7183	F = 0.23	*P* = 0.8761
**E2CpG15–16**	21	W = 0.961392	*P* = 0.5446	F = 0.98	*P* = 0.4234
**E2CpG18**	21	W = 0.852523	*P* = 0.0047	H = 2.7900	*P* = 0.4252
**E2CpG19**	21	W = 0.969337	P = 0.7183	F = 0.23	P = 0.8761

Note: ^a^ represents the number of samples analysed

All the CpG loci in the CpG island of Exon 2 were studied, and highly combined CpG loci were considered as one single locus. A total of 14 valid datasets of 17 CpG loci were obtained (Fig. 3c). Not all datasets distributed normally (Table 2). ANOVA or Kruskal–Wallis analysis showed that there was no significant difference in the methylation level of loci in relation to age (*P* > 0.05). However, the level of DNA methylation in the promoter was significantly lower than that in Exon 2 (T = 40.74, *P* < 0.001, Fig. 3d).

Sequence analysis showed that, although CpG island could be both detected in the promoter of chicken and human *KLF7* (supplementary Figure 1a), the DNA sequence similarity between them was low (supplementary Figure 1b). No homologous locus of CpG site was found in human *KLF7* promoter. However, the DNA sequence similarity between the Exon 2 in chicken and human *KLF7s* was high, and the loci of E2CpG1, E2CpG4, E2CpG7, E2CpG9 and E2CpG16 were conserved between chicken and human (supplementary Figure 1c).

### The association for DNA methylation with *KLF7* transcripts and blood metabolic indicators

**Fig. 4:**
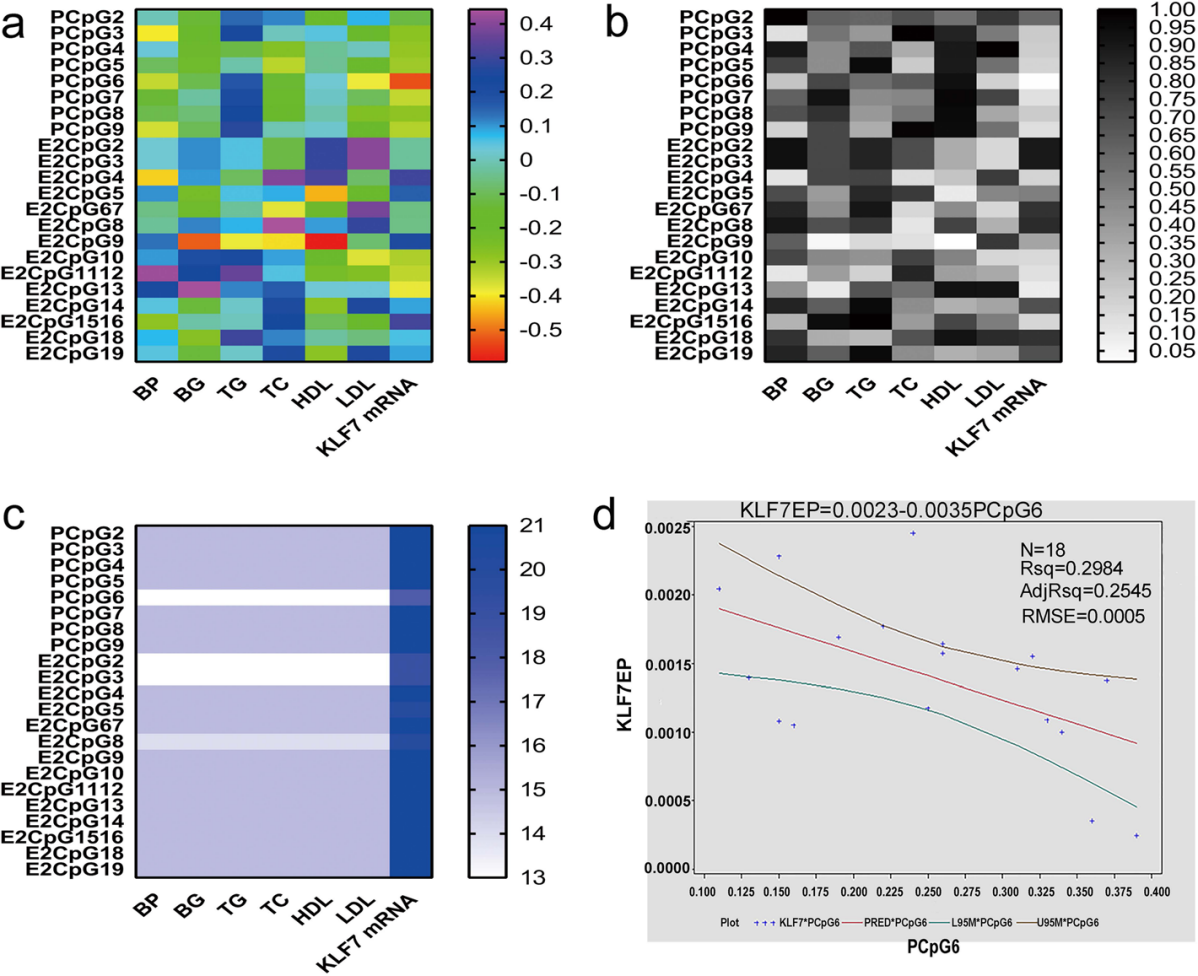
Association analysis of DNA methylation with *KLF7* transcripts and blood metabolic indicators. **a**. The r value of the Spearman correlation analysis. **b**. The *P* value of the Spearman correlation analysis. **c**. The number of samples used in the Spearman correlation analysis. **d**. The regression analysis between the DNA methylation of PCpG6 and *KLF7* transcripts in chicken adipose tissue

The association for the DNA methylation in each locus with *KLF7* transcripts was studied by Spearman correlation analysis. The results showed that the DNA methylation of PCpG6 was significantly correlated to *KLF7* transcripts (r = − 0.53099, *P* = 0.0234). However, no significant correlation between the DNA methylation of other loci and *KLF7* expression was found (*P* > 0.05, Fig. 4).

In addition, the DNA methylation of E2CpG9 was significantly correlated with fasting blood glucose level (r = − 0.51706, *P* = 0.0484) and blood HDL (r = − 0.59373, *P* = 0.0196). No other significant correlation between the DNA methylation of other loci and the blood metabolic indicators measured was found (P > 0.05, Fig. 4).

Both the dataset of PCpG6 DNA methylation and *KLF7* transcripts distributed normally (Shapiro–Wilk test, P > 0.05), so the regression relationship between PCpG6 DNA methylation and *KLF7* transcripts was studied using a generalized linear regression model. The established regression equation, Y (*KLF7* transcripts) = 0.00229–0.00351× (DNA methylation of PCpG6), was statistically significant (F = 6.80, *P* = 0.0190, R^2^ = 0.2545; Fig. 4d).

### Correlation between the DNA methylation of different CpG loci

The correlations between the methylation of different CpG loci were analyzed by Spearman correlation analysis. The results showed that there were significant associations for several CpG loci (*P* < 0.05). The methylation levels of PCpG3-PCpG9 were positively correlated with each other (*P* < 0.05), and the methylation level of PCpG2 was positively correlated with that of PCpG8 (*P* < 0.05). In addition, the methylation levels of PCpG4, PCpG5, and PCpG8 were significantly associated with that of E2CpG4 (P < 0.05). Additionally, the methylation data onto E2CpG2 and E2CpG14 were the same to those of E2CpG3 and E2CpG19, respectively (r = 1, *P* < 0.0001). The methylation data onto E2CpG2, E2CpG3, and E2CpG4 was negatively correlated with that of E2CpG11–12 (P < 0.05); The methylation data onto E2CpG4 was negatively correlated with that of E2CpG18 (P < 0.05), The methylation data onto E2CpG5 was positively correlated with that of E2CpG9 (P < 0.05), and the methylation data onto E2CpG8 was positively correlated with that of E2CpG13 (P < 0.05, Table 3).

**Table 3:** Correlation matrix of the CpG locus in the region of chicken *KLF7*

	**E2CpG2**	**E2CpG3**	**E2CpG4**	**E2CpG5**	**E2CpG6–7**	**E2CpG8**	**E2CpG9**	**E2CpG10**	**E2CpG11–12**	**E2CpG13**	**E2CpG14**
**E2CpG3**	1.0000 ^a^										
	<.0001 ^b^										
	19 ^c^										
**E2CpG4**	0.2938	0.2938									
	0.2221	0.2221									
	19	19									
**E2CpG5**	0.0197	0.0197	0.0859								
	0.9380	0.9380	0.7187								
	18	18	20								
**E2CpG67**	0.2824	0.2824	−0.3787	−0.0197							
	0.2414	0.2414	0.0905	0.9342							
	19	19	21	20							
**E2CpG8**	0.0366	0.0366	0.2197	0.2710	0.2730						
	0.8854	0.8854	0.3520	0.2618	0.2442						
	18	18	20	19	20						
**E2CpG9**	−0.1636	− 0.1636	− 0.0599	0.4711	0.3857	0.1230					
	0.5034	0.5034	0.7964	0.0360	0.0842	0.6054					
	19	19	21	20	21	20					
**E2CpG10**	−0.3801	− 0.3801	− 0.4156	− 0.0442	− 0.0668	− 0.0898	− 0.1541				
	0.1084	0.1084	0.0610	0.8532	0.7734	0.7065	0.5050				
	19	19	21	20	21	20	21				
**E2CpG11–12**	−0.4571	−0.4571	− 0.6615	0.2613	0.0946	0.2131	0.1588	0.4974			
	0.0491	0.0491	0.0011	0.2659	0.6834	0.3671	0.4918	0.0218			
	19	19	21	20	21	20	21	21			
**E2CpG13**	0.1260	0.1260	0.1022	0.0637	0.2334	0.7436	0.2532	−0.0261	0.3612		
	0.6072	0.6072	0.6595	0.7896	0.3087	0.0002	0.2680	0.9106	0.1077		
	19	19	21	20	21	20	21	21	21		
**E2CpG14**	0.3611	0.3611	0.0749	0.3268	0.3277	0.4237	0.3537	0.1157	0.1184	0.3634	
	0.1288	0.1288	0.7469	0.1597	0.1470	0.0627	0.1158	0.6175	0.6092	0.1054	
	19	19	21	20	21	20	21	21	21	21	
**E2CpG15–16**	0.0687	0.0687	0.4151	0.2157	0.0786	0.3225	0.3674	0.1090	−0.0307	0.3456	0.3779
	0.7800	0.7800	0.0613	0.3611	0.7348	0.1655	0.1013	0.6380	0.8950	0.1249	0.0912
	19	19	21	20	21	20	21	21	21	21	21
**E2CpG18**	−0.3398	−0.3398	− 0.4726	0.0556	0.2350	−0.0527	− 0.0214	0.1926	0.2877	−0.3586	− 0.1449
	0.1546	0.1546	0.0305	0.8160	0.3052	0.8255	0.9265	0.4030	0.2060	0.1104	0.5310
	19	19	21	20	21	20	21	21	21	21	21
**E2CpG19**	0.3611	0.3611	0.0749	0.3268	0.3277	0.4237	0.3537	0.1157	0.1184	0.3634	1.0000
	0.1288	0.1288	0.7469	0.1597	0.1470	0.0627	0.1158	0.6175	0.6092	0.1054	<.0001
	19	19	21	20	21	20	21	21	21	21	21
**PCpG2**	−0.3467	−0.3467	−0.2836	0.3315	−0.1760	0.0369	0.1558	0.1069	0.2605	−0.0689	−0.0848
	0.1459	0.1459	0.2129	0.1534	0.4454	0.8774	0.5002	0.6447	0.2542	0.7667	0.7149
	19	19	21	20	21	20	21	21	21	21	21
**PCpG3**	−0.2517	−0.2517	− 0.3798	− 0.1509	0.2249	− 0.0830	− 0.1594	0.2155	0.1652	− 0.2025	− 0.0603
	0.2987	0.2987	0.0895	0.5254	0.3271	0.7281	0.4900	0.3481	0.4742	0.3786	0.7953
	19	19	21	20	21	20	21	21	21	21	21
**PCpG4**	−0.4287	−0.4287	−0.4807	−0.2412	− 0.0719	−0.3458	− 0.0344	0.4087	0.1869	−0.2648	− 0.0884
	0.0670	0.0670	0.0274	0.3056	0.7566	0.1353	0.8825	0.0658	0.4172	0.2460	0.7032
	19	19	21	20	21	20	21	21	21	21	21
**PCpG5**	−0.4217	− 0.4217	−0.6114	− 0.2451	0.1056	−0.3069	− 0.1065	0.3589	0.2236	−0.3098	− 0.2323
	0.0722	0.0722	0.0032	0.2977	0.6488	0.1882	0.6460	0.1101	0.3300	0.1717	0.3108
	19	19	21	20	21	20	21	21	21	21	21
**PCpG6**	−0.3245	− 0.3245	− 0.2233	− 0.2761	0.0764	−0.1193	− 0.2020	0.4222	0.1364	−0.0861	− 0.1235
	0.2038	0.2038	0.3732	0.2833	0.7633	0.6482	0.4216	0.0809	0.5895	0.7341	0.6254
	17	17	18	17	18	17	18	18	18	18	18
**PCpG7**	−0.3755	−0.3755	− 0.5032	− 0.2610	0.0416	− 0.2866	− 0.2846	0.3672	0.1833	− 0.3242	− 0.4005
	0.1131	0.1131	0.0201	0.2663	0.8578	0.2206	0.2111	0.1015	0.4264	0.1516	0.0720
	19	19	21	20	21	20	21	21	21	21	21
**PCpG8**	−0.3497	−0.3497	−0.5527	− 0.0989	0.1695	−0.1919	− 0.0984	0.2266	0.3144	−0.2547	− 0.2960
	0.1422	0.1422	0.0094	0.6783	0.4626	0.4177	0.6714	0.3234	0.1652	0.2651	0.1926
	19	19	21	20	21	20	21	21	21	21	21
**PCpG9**	−0.2245	− 0.2245	− 0.4057	−0.1304	0.2498	− 0.0412	− 0.1825	0.2054	0.1496	−0.1909	− 0.1361
	0.3555	0.3555	0.0681	0.5838	0.2749	0.8632	0.4285	0.3719	0.5175	0.4072	0.5565
	19	19	21	20	21	20	21	21	21	21	21
	**E2CpG14**	**E2CpG15–16**	**E2CpG18**	**E2CpG19**	**PCpG2**	**PCpG3**	**PCpG4**	**PCpG5**	**PCpG6**	**PCpG7**	**PCpG8**
**E2CpG15–16**	0.3779										
	0.0912										
	21										
**E2CpG18**	−0.1449	−0.0417									
	0.5310	0.8577									
	21	21									
**E2CpG19**	1.0000	0.3779	−0.1449								
	<.0001	0.0912	0.5310								
	21	21	21								
**PCpG2**	−0.0848	−0.2857	0.1994	−0.0848							
	0.7149	0.2094	0.3863	0.7149							
	21	21	21	21							
**PCpG3**	−0.0603	−0.1634	0.5540	−0.0603	0.3050						
	0.7953	0.4792	0.0092	0.7953	0.1788						
	21	21	21	21	21						
**PCpG4**	−0.0884	−0.2127	0.2790	−0.0884	0.4169	0.6760					
	0.7032	0.3547	0.2207	0.7032	0.0601	0.0008					
	21	21	21	21	21	21					
**PCpG5**	−0.2323	−0.3160	0.4481	−0.2323	0.4101	0.7998	0.8982				
	0.3108	0.1628	0.0417	0.3108	0.0648	<.0001	<.0001				
	21	21	21	21	21	21	21				
**PCpG6**	−0.1235	−0.0142	0.3876	−0.1235	0.1251	0.9250	0.6821	0.7313			
	0.6254	0.9555	0.1120	0.6254	0.6208	<.0001	0.0018	0.0006			
	18	18	18	18	18	18	18	18			
**PCpG7**	−0.4005	−0.1929	0.5588	−0.4005	0.3459	0.7831	0.7776	0.8794	0.8090		
	0.0720	0.4022	0.0085	0.0720	0.1245	<.0001	<.0001	<.0001	<.0001		
	21	21	21	21	21	21	21	21	18		
**PCpG8**	−0.2960	−0.3326	0.5817	−0.2960	0.4773	0.8641	0.6400	0.8551	0.6924	0.7911	
	0.1926	0.1407	0.0057	0.1926	0.0287	<.0001	0.0018	<.0001	0.0015	<.0001	
	21	21	21	21	21	21	21	21	18	21	
**PCpG9**	−0.1361	−0.2181	0.5731	−0.1361	0.3592	0.9684	0.6016	0.7965	0.8591	0.7927	0.9092
	0.5565	0.3422	0.0066	0.5565	0.1098	<.0001	0.0039	<.0001	<.0001	<.0001	<.0001
	21	21	21	21	21	21	21	21	18	21	21

Note: ^a^, ^b^, and ^c^ represent the r value, *P* value and the number of samples analysed for the Spearman correlation analysis, respectively

### Principal component analysis of the methylation data

To avoid the misinterpretation of the interactions between different loci and the association of a single CpG locus with *KLF7* transcripts, the methylation data onto all CpG loci were subjected to principal component analysis (PCA). Fourteen effective principal components (z1–z14) were extracted from these 22 methylation data (Table 4).

**Table 4:** Principal Component Analysis of methylation data

	Eigenvalue	Difference	Proportion	Cumulative
**1**	8.58397411	4.89652080	0.3902	0.3902
**2**	3.68745330	1.31306607	0.1676	0.5578
**3**	2.37438723	0.54826523	0.1079	0.6657
**4**	1.82612200	0.30800856	0.0830	0.7487
**5**	1.51811344	0.34269849	0.0690	0.8177
**6**	1.17541496	0.21863636	0.0534	0.8712
**7**	0.95677860	0.22254876	0.0435	0.9146
**8**	0.73422984	0.26289337	0.0334	0.9480
**9**	0.47133647	0.24226708	0.0214	0.9694
**10**	0.22906939	0.03795582	0.0104	0.9799
**11**	0.19111357	0.03848379	0.0087	0.9885
**12**	0.15262978	0.07853660	0.0069	0.9955
**13**	0.07409317	0.04880904	0.0034	0.9989
**14**	0.02528414	0.02528414	0.0011	1.0000

### Factor analysis of new variables z1–z6

Six principal components (z1–z6) were extracted from 14 effective principal components by factor extraction (Eigenvalue > 1). The total variance contribution rate of the six new variables (z1–z6) was more than 85% of the total data. The new variables (z1–z6) obtained by PCA were subjected to factor analysis and named Factors 1–6. The results indicate that the loads of Factors 1–6 (z1–6) on each CpG locus were quite different. Factor 1 had positive loads on most loci in the promoter and negative loads on most loci in Exon 2 and had a higher load on the loci of PCpG3–PCpG9 and E2CpG4. Factor 2 had a high load on several loci in Exon 2, including E2CpG6–7, E2CpG8, E2CpG9, E2CpG13, E2CpG14, E2CpG15–16, and E2CpG19. Factor 3 had a high load on E2CpG2, E2CpG3, and E2CpG11–12. Factor 4 had a high load on E2CpG5. Factor 5 had a high load on PCpG2 (Table 5). Factor 6 had no obvious high load on any locus.

**Table 5:** Factor analysis of methylation data (Factor be retained by the MINEIGEN criterion)

	Factor1	Factor2	Factor3	Factor4	Factor5	Factor6
**PCpG5**	0.92834	0.11879	0.17878	0.10131	0.22061	0.03886
**PCpG7**	0.91141	0.03225	0.32208	− 0.00031	− 0.06598	− 0.02675
**PCpG8**	0.89855	0.10258	0.13508	−0.08460	0.17272	0.23556
**PCpG9**	0.85571	0.22213	0.33021	− 0.12622	0.12066	0.20036
**PCpG3**	0.84517	0.23869	0.31027	−0.08282	0.12230	0.11663
**PCpG4**	0.75354	0.08572	0.05237	0.29781	0.31393	−0.32154
**PCpG6**	0.73199	0.26981	0.49523	−0.02023	−0.07731	− 0.09970
**E2CpG10**	0.59539	0.24021	0.03243	0.30738	−0.48003	−0.34490
**E2CpG15–16**	−0.57427	0.52467	0.34678	0.08436	−0.28139	− 0.29456
**E2CpG4**	−0.84494	− 0.17986	0.16464	−0.09869	0.26624	−0.11090
**E2CpG14**	−0.34301	0.80962	0.00112	0.42052	0.04567	−0.13228
**E2CpG19**	−0.34301	0.80962	0.00112	0.42052	0.04567	−0.13228
**E2CpG13**	−0.29532	0.69629	−0.05477	−0.48668	0.35375	−0.13615
**E2CpG6–7**	−0.02327	0.65169	0.04899	−0.31275	− 0.19246	0.49352
**E2CpG9**	−0.24675	0.63233	−0.42568	0.25199	0.12345	0.40308
**E2CpG8**	−0.31118	0.58151	−0.04129	− 0.54707	0.14842	− 0.16275
**E2CpG2**	−0.59702	0.02401	0.71111	0.06984	−0.00995	0.14808
**E2CpG3**	−0.59702	0.02401	0.71111	0.06984	−0.00995	0.14808
**E2CpG11–12**	0.52934	0.34446	−0.53169	−0.32012	− 0.10485	−0.14235
**E2CpG5**	−0.27250	0.03287	−0.16176	0.57029	0.08492	0.39362
**PCpG2**	0.50184	−0.02296	−0.21338	0.31024	0.57542	−0.06843
**E2CpG18**	0.57945	0.17351	−0.25425	0.02533	−0.60630	0.15100

Factors were rotated by the *quartimax* method, and the results showed that Factor 1 mainly loads highly on the loci in the promoter, and Factors 2–6 load highly on the loci in Exon 2 (Table 6).

**Table 6:** Factor analysis of methylation data (Rotated Factor Pattern by Quartimax)

	Factor1	Factor2	Factor3	Factor4	Factor5	Factor6
**PCpG9**	0.96036	− 0.02374	− 0.08436	0.07716	0.11869	− 0.10607
**PCpG5**	0.94787	−0.16833	− 0.03008	− 0.08018	−0.16020	− 0.09249
**PCpG3**	0.94185	−0.04449	−0.04795	0.08003	0.05205	−0.05010
**PCpG7**	0.92477	−0.08229	− 0.20002	− 0.11142	− 0.00909	0.16138
**PCpG8**	0.90745	−0.19428	− 0.12816	− 0.02476	0.05945	− 0.21645
**PCpG6**	0.87870	0.11854	−0.02292	0.07802	0.02889	0.27673
**PCpG4**	0.72331	−0.22681	0.08899	−0.08498	− 0.52419	0.06339
**E2CpG4**	−0.71389	0.49487	−0.04194	0.22560	−0.20288	−0.13401
**E2CpG2**	−0.24561	0.89519	0.09107	0.03695	0.12860	0.04127
**E2CpG3**	−0.24561	0.89519	0.09107	0.03695	0.12860	0.04127
**E2CpG18**	0.40771	−0.51341	0.03221	−0.25990	0.47540	0.31167
**E2CpG11–12**	0.32049	−0.76497	0.02842	0.31551	0.13195	0.09815
**E2CpG14**	−0.10283	0.12752	0.92871	0.21458	−0.03918	0.18065
**E2CpG19**	−0.10283	0.12752	0.92871	0.21458	−0.03918	0.18065
**E2CpG9**	−0.16828	−0.22207	0.77846	0.07591	0.26525	−0.35127
**E2CpG5**	−0.23208	0.08102	0.46715	−0.45566	0.02841	−0.31904
**E2CpG13**	−0.10304	0.00649	0.33738	0.90219	0.04859	−0.12158
**E2CpG8**	−0.16643	−0.00372	0.19605	0.83363	0.15129	0.02258
**E2CpG6–7**	0.16568	0.02996	0.34104	0.36178	0.71717	−0.12719
**PCpG2**	0.42708	−0.29521	0.13624	−0.12019	−0.54346	− 0.36336
**E2CpG10**	0.52357	−0.28669	0.17966	−0.23173	−0.00468	0.64404
**E2CpG15–16**	−0.30850	0.44691	0.47334	0.30862	0.11909	0.51961

### Correlation and regression analysis of z1–z6 with *KLF7* expression and blood metabolic indexes

**Fig. 5:**
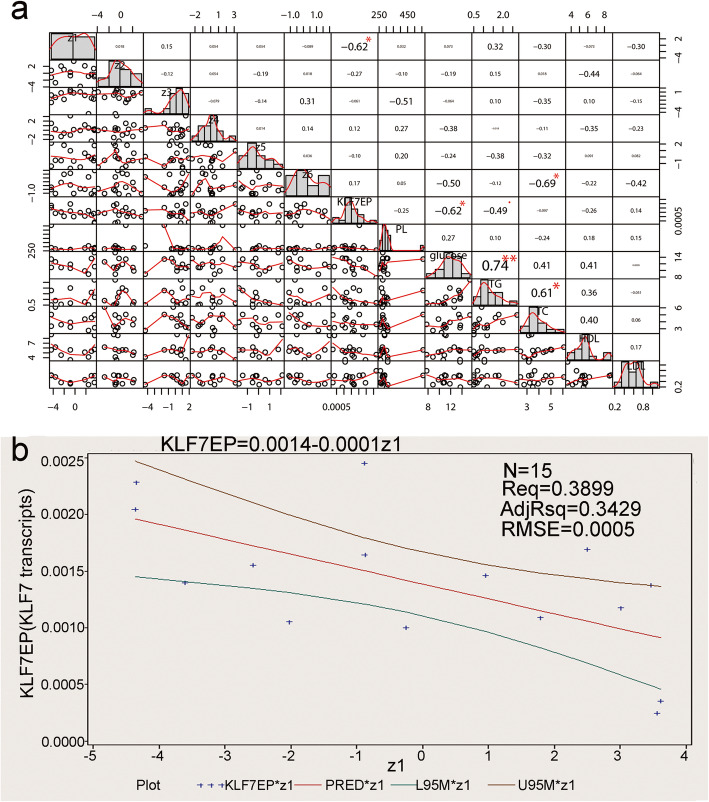
Association analysis of z1-z6 with *KLF7* transcripts and blood metabolic indicators. **a**. The Spearman correlation analysis of the z1–z6 with *KLF7* transcripts and blood metabolic indicators. **b**. The regression analysis between z1 and *KLF7* transcripts

Spearman correlation analysis showed that the new variable z1 was negatively correlated with *KLF7* transcripts (r = − 0.62439, *P* = 0.0128), while the new variables z2–z6 had no significant correlation with *KLF7* transcripts (*P* > 0.05). There was no significant correlation between z1–z6 and blood metabolic indexes but z6 was significantly correlated with TC level in the blood (Fig. 5a).

The data onto z1 and *KLF7* transcripts distributed normally (Shapiro–Wilk test, P > 0.05), and a regression relationship between z1 and *KLF7* transcripts was studied using generalized linear regression model. The results showed that the established regression equation was statistically significant (F = 8.31, P = 0.0128, R^2^ = 0.3429), and the regression equation was: Y (*KLF7* transcripts) = 0.00139–0.00013144 × z1 (Fig. 5b).

## Discussion

*KLF7* is a highly conserved gene in humans and animals [23, 24]. Previous reports on mammalian species showed that KLF7 regulated neuroectodermal and mesodermal development [2] and played a role in obesity [6], T2DM [7, 8], and blood disease [9]. Our previous study showed that KLF7 was an important regulator in chicken adipose tissue development [17, 18]. Currently, the results showed that *KLF7* transcripts of the adipose tissue of Chinese fast-growing yellow broilers were associated with age, in line with our previous report in white broilers [17]. Although there was no biologically significant difference in *KLF7* transcripts of the weeks of 2, 4, and 6, there was a downward trend at the week of 6. The decline in *KLF7* expression during the weeks of 2, 4, and 6 might be due to the suppression function of KLF7 in the formation of adipose tissue at the early stage [17]. In addition, the increase in *KLF7* transcripts at the age of 8 weeks suggested that chicken KLF7 might have a function in mature adipose tissue, similar to the report on its orthology in human in vitro [7, 8].

**Fig. 6:**
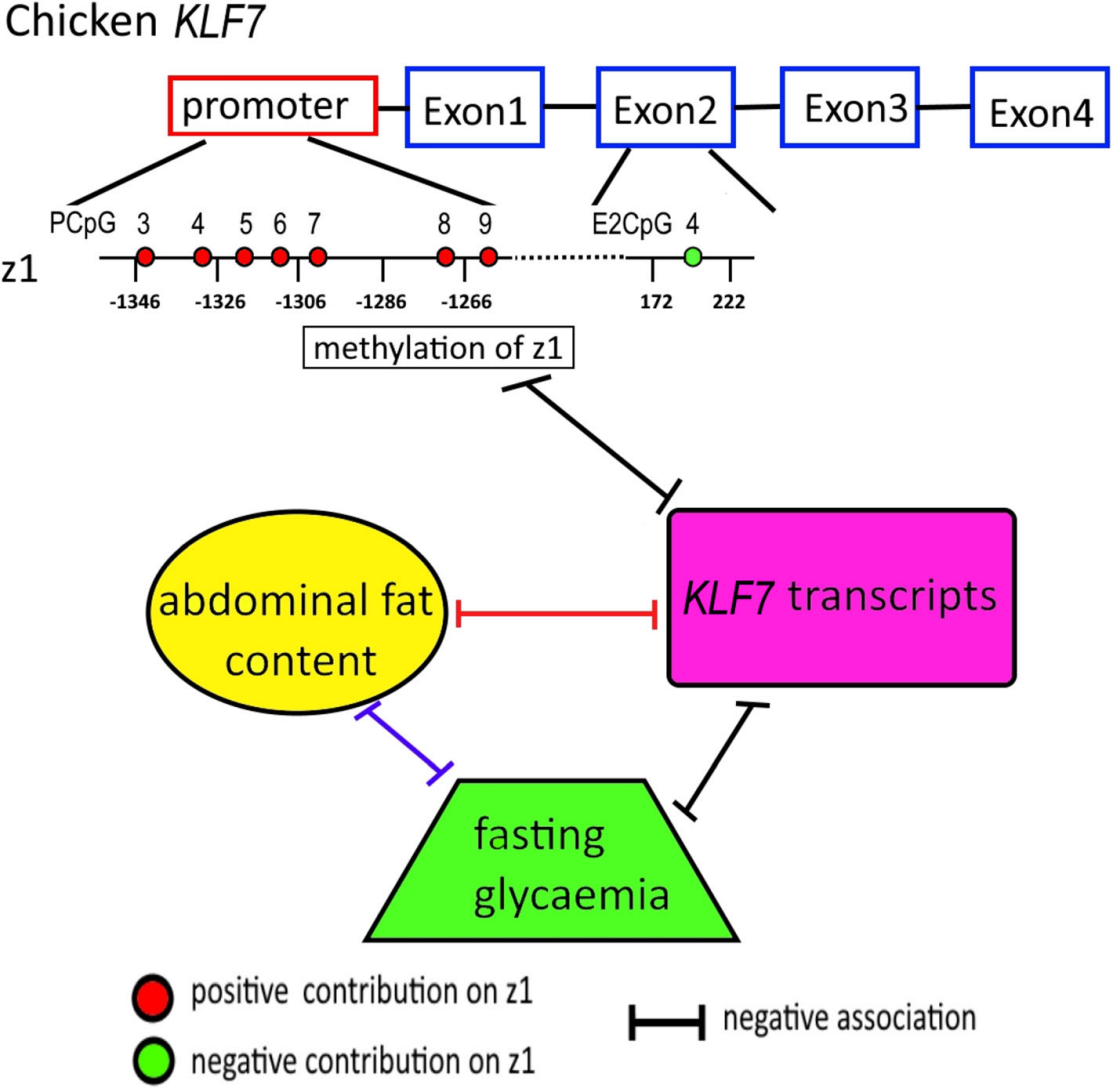
Schematic representation of the relationship of DNA methylation, *KLF7* transcripts, glycaemia and abdominal fat content in chicken. The negative associations marked red and blue were confirmed by the reference [17, 25], respectively

The level of blood glucose was changeable and associated with insulinaemia in chicken [25]. Here, the results showed that chicken *KLF7* transcripts were correlated with fasting blood glucose level, in line with our previous reports in white broilers that the chicken *KLF7* was involved in the regulation of adipogenesis and blood metabolic indicators [17, 18], and provided additional evidence for the role of KLF7 in metabolic syndrome from the perspective of non-rodent model animals. In addition, the previous study showed that chickens selected for low fasting glycaemia (LG) were fattier than their counterparts selected for high fasting glycaemia (HG) [25]. The negative correlation of *KLF7* expression to the fasting glycaemia in chicken and abdominal fat content [17, 18], suggested that a feedback regulation among *KLF7* expression, glycaemia, and obesity might exist in chicken (Fig. 6).

DNA methylation is important to the regulation of gene expression and function in animals [19]. The previous studies in humans showed that DNA methylation of *KLF7* was associated with the occurrence and development of gastric cancer [20, 21], however, there is no report on the DNA methylation of *KLF7* in adipose tissue and birds. Sequence analysis showed that there was a similar distribution of CpG-rich sequence in chicken *KLF7* as that of human *KLF7*, suggested that the chicken *KLF7* might be also regulated by DNA methylation.

Sequenom MassArray were used to study the DNA methylation in the promoter and Exon 2 of chicken *KLF7*. A total of 22 valid datasets were obtained, and the level of DNA methylation in the promoter was lower than those in Exon 2. This was probably because it is a consecutively expressed gene during adipogenesis [17]; therefore, the promoter of chicken *KLF7* could not be strongly silenced by a long-term mechanism like DNA methylation in adipose tissue.

In addition, none of the loci detected were significantly different among the ages of 2, 4, 6, and 8 weeks, indicated that the DNA methylation might not be a main regulation method of the *KLF7* expression in adipose tissue during development.

The association analysis showed that only the methylation of PCpG6 was significantly associated with *KLF7* transcripts in chicken abdominal adipose tissue, and the contribution of PCpG6 to the variation on *KLF7* transcripts was 0.2545. Sequence analysis showed that there were several binding sites of transcriptional factors at the locus of PCpG6, including TFAP2C and TFAP2A (supplementary Table 1), The negative correlation between DNA methylation of PCpG6 with *KLF7* transcripts might be mediated by transcriptional factor. However, further investigation is needed to verify this hypothesis.

Our previous study showed that one SNP (c. A141G) in the *KLF7* coding sequence was associated with blood very-low-density lipoprotein and abdominal fat content in broilers [18] and chicken KLF7 regulated the promoter activity of lipoprotein lipase (LPL) [17]. In the current study, the result showed that the methylation of E2CpG9 was significantly associated with blood HDL level, further suggested that KLF7 might play a role in the fat transport in chicken. In addition, the E2CpG9 was conserved between chicken and human *KLF7s* (supplementary Figure 1C), this result might provide a clue to the function of KLF7 in human.

To avoid the misinterpretation of the interaction effect of different loci and to discover the relationship between general DNA methylation and *KLF7* transcripts, the methylation data were subjected to PCA. Fourteen effective principal components (z1–z14) were extracted from these 22 methylation data. Six principal components (z1–z6) were extracted from 14 principal components by factor extraction, and named Factors 1–6, respectively.

Factor analysis showed that Factor 1 had a higher load on the loci of PCpG3–PCpG9 and E2CpG4, indicating that Factor 1 (z1) mainly represents the effect of DNA methylation in the promoter. Factors 2–6 loaded highly with quite different loci in Exon 2 and PCpG2. Therefore, Factors 2–6 might represent the effect of DNA methylation in Exon 2, and there was a large difference in them.

Correlation analysis showed that the new variable z1 was negatively correlated with *KLF7* transcripts, whereas none of these z2–z6 were significant correlated with *KLF7* transcripts. In addition, the regression relationship between z1 and *KLF7* transcripts was studied, and the contribution of z1 to the variation on *KLF7* transcripts was 0.3429, which was greater than the contribution of the single locus PCpG6. Additionally, the ratio of the slope to truncation was about 9.5%, indicating the greatest effect that Factor 1 (z1) had on the *KLF7* transcripts was about 9.5%. This was reasonable for effect of DNA methylation on gene expression, indicated that the *KLF7* transcripts of chicken abdominal adipose tissue might be inhibited by DNA methylation in promoter. Sequence analysis showed that there were many binding sites of transcription factors at the loci of PCpG3–PCpG9 in chicken *KLF7* promoter, respectively (supplementary Table 1), The inhibitory effect of DNA methylation on *KLF7* expression might be achieved in part by affecting the binding of transcription factors to the *KLF7* promoter, like the report on chicken *ApoA-I* [26].

There was no significant correlation between z1 and blood metabolic indexes. This might be because DNA methylation in chicken *KLF7* does not directly take part in the regulation of blood metabolic indexes. However, further investigation is needed into whether an indirect association exists on DNA methylation in chicken *KLF7* and blood metabolic indexes.

## Conclusions

In conclusion, the *KLF7* transcripts of chicken abdominal adipose tissue might be inhibited by DNA methylation in the promoter, and it might be related to the DNA methylation level of PCpG6 (Fig. 6).

## Methods

### Experimental birds and management

Animal work was conducted according to the guidelines on the care and use of experimental animals established by the Ministry of Science and Technology of the People’s Republic of China (approval number 2006–398) and was approved by the Animal Experimental Ethical Committee of the First Affiliated Hospital, Shihezi University School of Medicine. A total of 21 one-day-old male Chinese fast-growing yellow broilers were used in the current study, they were obtained from the Guangdong Wiz Agricultural Science & Technology Co. Ltd. (Guangzhou, China). All birds were kept in similar environmental conditions and had free access to feed and water until sacrificed.

### Tissues

Several male birds (*n* = 4–6) were sacrificed 6 h after a meal at 2, 4, 6, and 8 weeks of age by cutting the carotid artery after ether anaesthesia, and the abdominal fat tissue was collected immediately. The collected tissues were flash-frozen and stored in liquid nitrogen until the extraction of RNA. At the age of 4, 6, and 8 weeks, the peripheral blood was also collected from wing vein to measure blood metabolic indicators.

### RNA isolation

Total RNA of abdominal fat tissue (each 100 mg) was extracted using Trizol (Invitrogen) following the manufacturer’s protocol.

### RT-qPCR

The RNA quality was assessed by denaturing formaldehyde agarose gel electrophoresis. Reverse transcription was performed using 1 μg of total RNA, an oligo (dT) anchor primer, and ImProm-II reverse transcriptase (Promega, Madison, USA). Reverse transcription conditions for each cDNA amplification were 25 °C for 5 min, 42 °C for 60 min, and 70 °C for 15 min. Real-time quantitative PCR (qPCR) was used to detect *KLF7* and *β-ACTIN* using SYBR Premix Ex Taq (Takara) and a 7500 Real-Time PCR System (Applied Biosystems, Foster City, USA). Chicken *β-ACTIN* was used as an internal reference. The primers used are presented in Table 7. Samples (1 μL) from each reverse transcription reaction product were amplified in a 20-μl. PCR reaction. Reaction mixtures were incubated in an ABI Prism 7500 sequence detection system (Applied Biosystems) programmed for 1 cycle at 95 °C for 30 s and 40 cycles at 95 °C for 5 s, and at 60 °C for 34 s. Dissociation curves were analyzed using Dissociation Curve 1.0 software (Applied Biosystems) for each PCR to detect and eliminate possible primer–dimer artifacts. All reactions were performed in triplicate. The relative amounts of the *KLF7* transcripts were calculated by the comparative cycle-time method.

**Table 7:** Oligonucleotides used in the current study

Gene	Application	Primer (5′-3′)
*β-ACTIN*	*RT-qPCR*	F:CCTGGCACCTAGCACAATGA
		R:CCTGCTTGCTGATCCACATC
*KLF7*	*RT-qPCR*	F:TGCCATCCTTGGAGGAGAAC
		R:AGGCATGAAGGAAGCAGTCC
KLF7	*methylation analysis in promoter*	F: aggaagagagTTAGTTTGTTTTTGTTGTTGTGGAG
		R:cagtaatacgactcactatagggagaaggctAAATTACCTTTTCCAAAACTAAATCC
KLF7	*methylation analysis in Exon 2*	F:aggaagagagTGGATTTTATTTTTTTGTTAGTGGAG
		R:cagtaatacgactcactatagggagaaggctATTTTCTAAAAATACTTCCAATCCC

### Sequenom MassARRAY methylation analysis

Sequenom MassARRAY methylation analysis was carried out by Beijing Compass Biotechnology Co., Ltd. (Beijing, China). Briefly, DNA from adipose tissues was isolated using a QIAamp DNA Mini Kit (Qiagen, Hilden, Germany) following the manufacturer’s instruction. Genomic DNA quantification was detected by NanoDrop spectrophotometer (GE Healthcare Life Science, Uppsala, Sweden), then bisulfite conversation of the DNA was performed using EpiTect Bisulfite Kit (Qiagen) according to the manufacturer’s protocol. Two sets of primers were designed by EpiDesigner software (http://epidesigner.com; Table 7). The methylation level of individual units was measured by Quantitative Methylation Analysis (MassARRAY EpiTYOER, Sequenom, San Diego, CA). The data of MassARRAY methylation can be achieved in Supplementary Table 2.

### Bioinformatics analysis

The CpG density of the genomic region of chicken *KLF7* and human *KLF7* analyzed by CpGplot (Version 6.6.0). The genomic sequence of chicken *KLF7* was obtained from the NCBI and UCSC databases referenced to the promoter sequence (GenBank accession number: JX290203) and the full-length coding sequence of chicken *KLF7* (GenBank accession number: JQ736790). The sequence analyzed was 64,584 bp in length and included the 3000 bp sequence upstream of the predicted transcription start site and the 100 bp sequence downstream of the predicted 3′UTR of chicken *KLF7*. The pattern of transcriptional factor binding sites in DNA sequence was analyzed by JASPAR2020.

### Statistical analysis

All statistical analyses were performed using the SAS software system (version 9.2; SAS Inst. Inc., Cary, NC, USA). The Shapiro–Wilk test was used to test the normality of data. The difference in groups was analyzed by ANOVA or Kruskal–Wallis tests. The comparison of data was performed by PROC GLM procedure followed by Duncan’s multiple tests. With the following models:

$$ Y=\mu +\mathrm{F}+\mathrm{e} $$

where Y is the dependent variable, μ is the population mean, F is the fixed effect of age and e is the random error.

The data that conformed to the normal distribution was directly applied to the models, and the data that did not conform to the normal distribution were sorted rank firstly, and the ranks were then applied to the models. The correlation was analyzed by Spearman’s correlation test. The unpaired student *t* test was used to analyze the difference between two groups. The PCA and factor analysis was conducted by the PROC PRINCOMP and FACTOR procedure, respectively. Linear regression is performed with the PROG REG command of SAS. The *P*-value < 0.05 was considered statistically significant.

## Supplementary information

**Additional file 1: Supplementary Figure 1.** The sequence analysis of chicken and human *KLF7s*. a. The CpG density in the genomic region of human *KLF7* analyzed by CpGplot (Version 6.6.0). b. The dot plot analysis between the sequence studied for DNA methylation in chicken *KLF7* promoter and human *KLF7* promoter (3000 bp upstream of NM_003709.4). c. The alignment of the sequences of Exon 2 between chicken and human *KLF7s*.

**Additional file 2: Supplementary Table 1.** The prediction of transcription factor binding sites at the CpG loci in chicken *KLF7* promoter (analyzed using JASPAR2020, Search profile = ChIP-seq, Relative profile score threshold = 0.8). **Supplementary Table 2.** The DNA methylation data of CpG loci in the chicken *KLF7* gene detected by Sequenom MassArray. **Supplementary Table 3.** The mRNA expression data of *KLF7* in adipose tissue and phenotype data.

## Data Availability

The datasets analysed during the current study are available in the supplementary Table 2 and 3.

## Abbreviations

ANOVA: Analysis of variance
GLM: A general linear model
PCA: Principal component analysis
qRT-PCR: Quantitative real-time PCR
SNP: Single nucleotide polymorphism
Glu: Glucose
PL: Phospholipids
TC: Total cholesterol
TG: Triglyceride
LDL: Low-density lipoprotein
HDL: High-density lipoprotein
KLF7: Krüppel-like factor 7
UTR: Untranslated Region
