# Supplementary figures and images for: DNA methylation of CpG sites in the chicken KLF7 promoter and Exon 2 in association with mRNA expression in abdominal adipose tissue and blood metabolic indicators
Source: BMC Genet. 2020 Oct 14;21:120. doi: 10.1186/s12863-020-00923-6 (PMC7558735; doi:10.1186/s12863-020-00923-6)

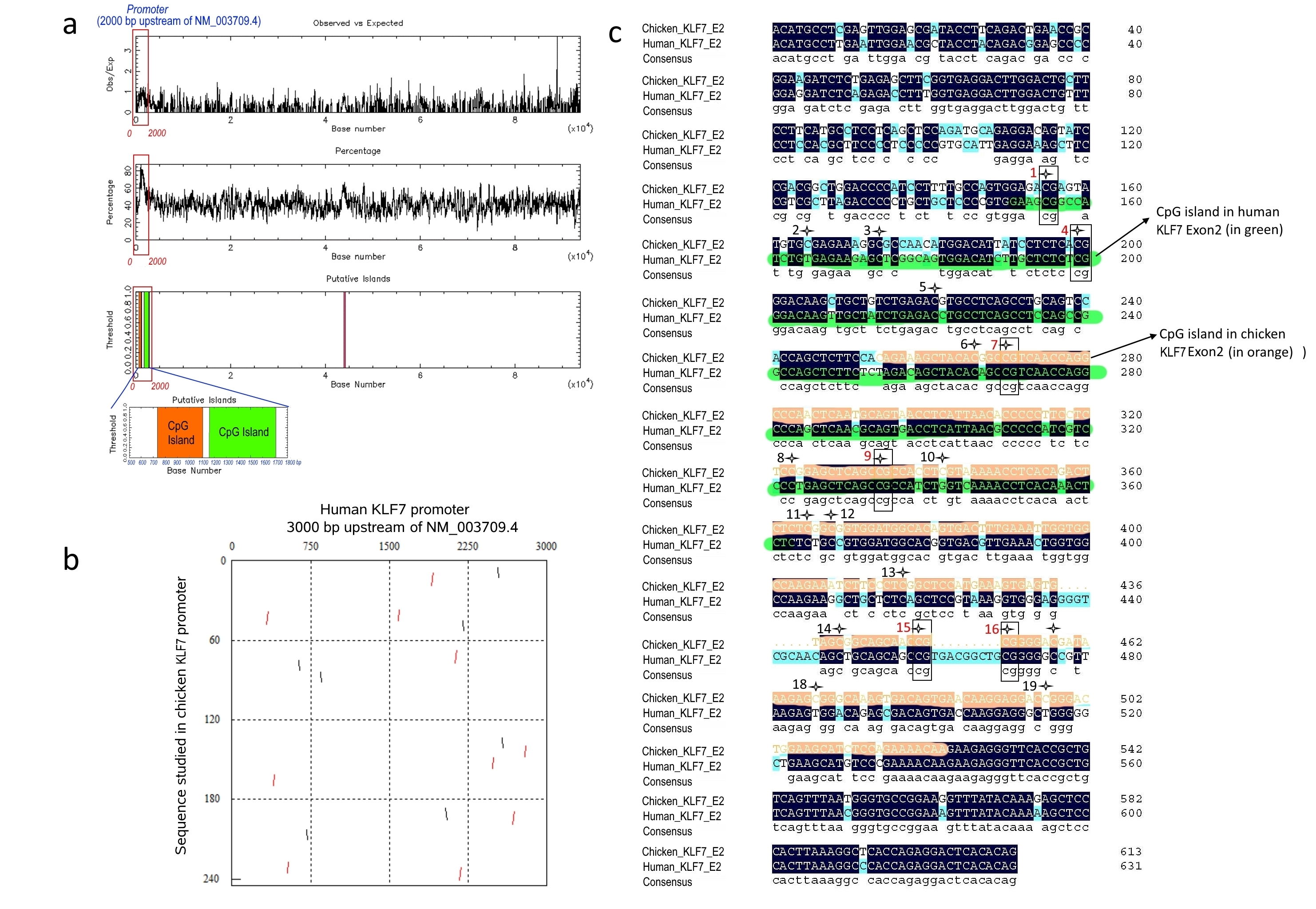

Supplement: Supplementary file 1 — Additional file 1: Supplementary Figure 1. The sequence analysis of chicken and human KLF7s. a. The CpG density in the genomic region of human KLF7 analyzed by CpGplot (Version 6.6.0). b. The dot plot analysis between the sequence studied for DNA methylation in chicken KLF7 promoter and human KLF7 promoter (3000 bp upstream of NM_003709.4). c. The alignment of the sequences of Exon 2 between chicken and human KLF7s. [file 12863_2020_923_MOESM1_ESM.jpg]
