# Supplementary material for: DNA methylation of CpG sites in the chicken KLF7 promoter and Exon 2 in association with mRNA expression in abdominal adipose tissue and blood metabolic indicators
Source: BMC Genet. 2020 Oct 14;21:120. doi: 10.1186/s12863-020-00923-6 (PMC7558735; doi:10.1186/s12863-020-00923-6)
Supplement: Supplementary file 2 — Additional file 2: Supplementary Table 1. The prediction of transcription factor binding sites at the CpG loci in chicken KLF7 promoter (analyzed using JASPAR2020, Search profile = ChIP-seq, Relative profile score threshold = 0.8). Supplementary Table 2. The DNA methylation data of CpG loci in the chicken KLF7 gene detected by Sequenom MassArray. Supplementary Table 3. The mRNA expression data of KLF7 in adipose tissue and phenotype data. [file 12863_2020_923_MOESM2_ESM.docx]

**Supplementary Table 1.** The prediction of transcription factor binding sites at the CpG loci in chicken *KLF7* promoter (analyzed using JASPAR2020, Search profile=ChIP-seq, Relative profile score threshold=0.8).

| **Locus** | Transcription factor binding sites |
| --- | --- |
| **PCpG2** | TCF3，TCF12，ZEB1，ELF1， ZBTB7A，FLI1，Gabpa |
| **PCpG3** | ZFP57，NFYB，KLF5，KLF15，SP1，SP2，HAP1，NFYC，NFYA |
| **PCpG4** | MAFK，Myc，RBPJ，FOXP1，FOXK1，FOXP2，Prdm15，FOXK2 |
| **PCpG5** | FOXP1，FOXK1，FOXP2，Prdm15，FOXK2，Bhlhe40，Myc，HIF1A，TFAP2C，TFAP2A，ZBTB14，NRF1 |
| **PCpG6** | TFAP2C，TFAP2A，ZBTB14，NRF1， |
| **PCpG7** | Zfx，TFAP2A，Tcfcp2l1 |
| **PCpG8** | NRF1，TFAP2C，TFAP2A，Zfx，Tcfcp2l1 |
| **PCpG9** |  |

**Supplementary table 2** The DNA methylation data of CpG loci in the chicken *KLF7* gene detected by Sequenom MassArray

| **Obs** | **age** | PCpG2 | PCpG3 | PCpG4 | PCpG5 | PCpG6 | PCpG7 | PCpG8 | PCpG9 | E2CpG2 | E2CpG3 | E2CpG4 | E2CpG5 | E2CpG67 | E2CpG8 | E2CpG9 | E2CpG10 | E2CpG1112 | E2CpG13 | E2CpG14 | E2CpG1516 | E2CpG18 | E2CpG19 |
| --- | --- | --- | --- | --- | --- | --- | --- | --- | --- | --- | --- | --- | --- | --- | --- | --- | --- | --- | --- | --- | --- | --- | --- |
| **1** | **2weeks** | 0.09 | 0.23 | 0.13 | 0.1 | 0.31 | 0.09 | 0.18 | 0.28 | 0.9 | 0.9 | 0.28 | 0.75 | 0.83 | 0.75 | 0.75 | 0.71 | 0.86 | 0.88 | 0.8 | 0.91 | 0.99 | 0.8 |
| **2** | **2weeks** | 0.1 | 0.26 | 0.17 | 0.11 | 0.34 | 0.07 | 0.19 | 0.3 | 0.95 | 0.95 | 0.18 | 0.63 | 0.92 | 0.81 | 0.98 | 0.76 | 0.91 | 0.95 | 0.95 | 0.94 | 0.98 | 0.95 |
| **3** | **2weeks** | 0.18 | 0.22 | 0.13 | 0.11 | 0.22 | 0.07 | 0.19 | 0.27 | 0.59 | 0.59 | 0.14 | NA | 0.89 | 0.82 | 0.89 | 0.78 | 0.94 | 0.91 | 0.89 | 0.93 | 0.98 | 0.89 |
| **4** | **2weeks** | 0.23 | 0.33 | 0.18 | 0.15 | 0.37 | 0.11 | 0.23 | 0.36 | 0.62 | 0.62 | 0.19 | 0.68 | 0.88 | 0.81 | 0.86 | 0.75 | 0.92 | 0.91 | 0.91 | 0.92 | 1 | 0.91 |
| **5** | **2weeks** | 0.24 | 0.22 | 0.09 | 0.1 | 0.16 | 0.05 | 0.19 | 0.28 | 0.92 | 0.92 | 0.46 | 0.83 | 0.91 | 0.85 | 1 | 0.6 | 0.88 | 0.95 | 0.89 | 0.91 | 0.97 | 0.89 |
| **6** | **2weeks** | 0.3 | 0.19 | 0.09 | 0.07 | NA | 0.07 | 0.16 | 0.26 | 0.6 | 0.6 | 0.28 | 0.73 | 0.85 | 0.78 | 0.97 | 0.65 | 0.88 | 0.93 | 0.84 | 0.93 | 0.98 | 0.84 |
| **7** | **4weeks** | 0.08 | 0.25 | 0.17 | 0.16 | 0.26 | 0.1 | 0.22 | 0.31 | NA | NA | 0.16 | 0.53 | 0.89 | 0.75 | 0.88 | 0.64 | 0.86 | 0.9 | 0.84 | 0.92 | 0.99 | 0.84 |
| **8** | **4weeks** | 0.08 | 0.27 | 0 | 0.08 | 0.26 | 0.07 | 0.22 | 0.34 | 1 | 1 | 0.27 | 0.76 | 0.91 | 0.81 | 0.85 | 0.64 | 0.89 | 0.91 | 0.88 | 0.93 | 1 | 0.88 |
| **9** | **4weeks** | 0.4 | 0.3 | 0.16 | 0.14 | 0.33 | 0.09 | 0.22 | 0.37 | 0.91 | 0.91 | 0.15 | 0.85 | 0.87 | 0.85 | 0.87 | 0.74 | 0.92 | 0.93 | 0.93 | 0.92 | 1 | 0.93 |
| **10** | **4weeks** | 0.14 | 0.12 | 0.05 | 0.05 | 0.13 | 0.03 | 0.11 | 0.18 | 0.96 | 0.96 | 0.77 | 0.73 | 0.84 | 0.78 | 0.79 | 0.69 | 0.86 | 0.9 | 0.87 | 0.91 | 0.97 | 0.87 |
| **11** | **4weeks** | 0.31 | 0.24 | 0.13 | 0.11 | 0.19 | 0.06 | 0.24 | 0.3 | 0.51 | 0.51 | 0.07 | 0.79 | 0.88 | 0.75 | 1 | 0.69 | 0.94 | 0.9 | 0.86 | 0.88 | 1 | 0.86 |
| **12** | **6weeks** | 0.21 | 0.05 | 0.05 | 0.04 | 0.11 | 0.04 | 0.11 | 0.15 | 0.95 | 0.95 | 0.5 | 0.82 | 0.88 | 0.83 | 0.96 | 0.67 | 0.87 | 0.91 | 0.94 | 0.94 | 1 | 0.94 |
| **13** | **6weeks** | 0.36 | 0.34 | 0.18 | 0.15 | 0.39 | 0.1 | 0.3 | 0.41 | 0.82 | 0.82 | 0.22 | 0.75 | 0.82 | 0.81 | 0.8 | 0.68 | 0.91 | 0.91 | 0.86 | 0.9 | 0.98 | 0.86 |
| **14** | **6weeks** | 0.35 | 0.26 | 0.13 | 0.13 | 0.25 | 0.1 | 0.26 | 0.36 | 0.73 | 0.73 | 0.15 | 0.5 | 0.9 | 0.83 | 0.79 | 0.74 | 0.91 | 0.93 | 0.84 | 0.91 | 1 | 0.84 |
| **15** | **6weeks** | 0.28 | 0.32 | 0.16 | 0.17 | 0.36 | 0.12 | 0.28 | 0.42 | 0.96 | 0.96 | 0.13 | 0.68 | 0.93 | 0.75 | 0.87 | 0.7 | 0.86 | 0.89 | 0.86 | 0.89 | 1 | 0.86 |
| **16** | **6weeks** | 0.22 | 0.13 | 0.07 | 0.06 | 0.15 | 0.05 | 0.14 | 0.23 | 1 | 1 | 0.12 | 0.78 | 0.92 | NA | 0.88 | 0.68 | 0.92 | 0.94 | 0.93 | 0.9 | 0.97 | 0.93 |
| **17** | **6weeks** | 0.24 | 0.21 | 0.11 | 0.1 | NA | 0.08 | 0.21 | 0.28 | 0.92 | 0.92 | 0.25 | 0.87 | 0.87 | 0.82 | 0.9 | 0.77 | 0.94 | 0.95 | 0.86 | 0.95 | 0.98 | 0.86 |
| **18** | **8weeks** | 0.04 | 0.25 | 0.03 | 0.06 | 0.32 | 0.05 | 0.19 | 0.32 | 0.91 | 0.91 | 0.8 | 0.76 | 0.92 | 0.88 | 0.94 | 0.68 | 0.86 | 0.94 | 0.91 | 0.94 | 0.98 | 0.91 |
| **19** | **8weeks** | 0.43 | 0.27 | 0.14 | 0.11 | NA | 0.09 | 0.22 | 0.33 | NA | NA | 0.2 | 0.89 | 0.92 | 0.82 | 1 | 0.66 | 0.88 | 0.9 | 0.88 | 0.93 | 1 | 0.88 |
| **20** | **8weeks** | 0.34 | 0.24 | 0.14 | 0.11 | 0.24 | 0.07 | 0.21 | 0.3 | 0.95 | 0.95 | 0.59 | 0.79 | 0.86 | 0.72 | 0.91 | 0.7 | 0.79 | 0.89 | 0.92 | 0.93 | 0.97 | 0.92 |
| **21** | **8weeks** | 0.22 | 0.14 | 0.08 | 0.06 | 0.15 | 0.04 | 0.13 | 0.21 | 0.98 | 0.98 | 1 | 0.66 | 0.87 | 0.84 | 0.85 | 0.51 | 0.85 | 0.94 | 0.88 | 0.92 | 0.96 | 0.88 |

**Supplementary table 3** The mRNA expression data of *KLF7* in adipose tissue and phenotype data

| **Obs** | **age** | KLF7EP | PL | Glu | TG | TC | HDL | LDL |
| --- | --- | --- | --- | --- | --- | --- | --- | --- |
| **1** | **2weeks** | 0.001459005 | NA | NA | NA | NA | NA | NA |
| **2** | **2weeks** | 0.001001936 | NA | NA | NA | NA | NA | NA |
| **3** | **2weeks** | 0.001771504 | NA | NA | NA | NA | NA | NA |
| **4** | **2weeks** | 0.001373505 | NA | NA | NA | NA | NA | NA |
| **5** | **2weeks** | 0.001051487 | NA | NA | NA | NA | NA | NA |
| **6** | **2weeks** | 0.001420033 | NA | NA | NA | NA | NA | NA |
| **7** | **4weeks** | 0.001572537 | 280.6613406 | 10.41 | 0.49 | 2.46 | 5.33 | 0.56 |
| **8** | **4weeks** | 0.00164134 | 266.7271123 | 11.86 | 2 | 3.92 | 5.61 | 0.32 |
| **9** | **4weeks** | 0.001088389 | 283.120322 | 9.84 | 0.78 | 4.39 | 5.13 | 0.46 |
| **10** | **4weeks** | 0.001397138 | 272.4647357 | 12.62 | 0.9 | 6.09 | 6.18 | 0.56 |
| **11** | **4weeks** | 0.00169028 | 310.169118 | 8.85 | 0.51 | 2.76 | 4.16 | 0.28 |
| **12** | **6weeks** | 0.002044749 | 559.3459055 | 13.46 | 1.78 | 5.05 | 5.62 | 0.62 |
| **13** | **6weeks** | 0.000246235 | 275.7433777 | 15.44 | 2.72 | 4.98 | 5.71 | 0.36 |
| **14** | **6weeks** | 0.001174118 | 274.9237172 | 13.65 | 1.61 | 4.58 | 5.34 | 0.61 |
| **15** | **6weeks** | 0.000350903 | 288.8579454 | 11.16 | 1.19 | 3.15 | 8.59 | 0.61 |
| **16** | **6weeks** | 0.001079723 | 310.169118 | 13.65 | 1.05 | 3.15 | 3.58 | 1.11 |
| **17** | **6weeks** | 0.000897075 | 302.7921737 | 13.72 | 1.19 | 3.49 | 5.5 | 0.19 |
| **18** | **8weeks** | 0.001551345 | 254.432205 | 10.87 | 0.57 | 3.6 | 4.54 | 0.36 |
| **19** | **8weeks** | 0.003180678 | 259.350168 | 7.75 | 0.69 | 3.65 | 4.63 | 0.79 |
| **20** | **8weeks** | 0.00245111 | 260.1698284 | 10.69 | 0.67 | 3.22 | 3.55 | 0.35 |
| **21** | **8weeks** | 0.002282236 | 288.8579454 | 10.92 | 0.5 | 3.38 | 8.77 | 0.63 |
